# Response to: The Importance of Conscious Sedation for Life-Saving Valve Procedures in Patients With Rheumatic Heart Disease From Low- to Middle-Income Countries

**DOI:** 10.5334/gh.377

**Published:** 2020-02-06

**Authors:** Melissa Persad, Jonathan Lazari, Mahmood Ahmad

**Affiliations:** 1 Department of Cardiology, Royal Free Hospital, London, UK

**Keywords:** Mitral stenosis, percutaneous mitral commissurotomy, conscious sedation, health economics

## Abstract

A response to Alcici et al., 2019 – thinking about how using conscious sedation for percutaneous mitral commissurotomy may improve outcomes in other areas.

We read with interest the study by Alcici et al. [1] demonstrating that conscious sedation using midazolam and fentanyl for percutaneous mitral commissurotomy (PMC) is safe, effective and practical. Rheumatic heart disease (RHD) remains a significant contributor to morbidity and mortality, particularly in low- and middle-income countries. As such, we welcome these results which may help improve access to potentially life-saving procedures such as PMC, especially in places where the cost and availability of anaesthetic drugs may be a barrier, as the method of sedation used led to no significant haemodynamic or intra-cardiac pressure changes on invasive measurements. We considered in whom these results could be expanded.

For example in pregnant patients requiring PMC for severe mitral stenosis (MS). Firouzi et al. [2] carried out PMC via the Inoue technique in 31 pregnant women with significant MS. The procedural success rate was 93.5%, with no maternal deaths or acute pulmonary odema. The mean mitral valve area (MVA) significantly increased post-procedure (from 0.73 ± 0.17 cm^2^ to 1.28 ± 0.24 cm^2^; P < 0.001). It may be plausible that conscious sedation may be a way to further improve outcomes. Specifically, given how high risk severe MS can be in pregnancy, the low haemodynamic impact of conscious sedation could be considered to improve safety and outcomes in this population with these widely available anaesthetic drugs. Especially as this population presents late in developed countries in areas where anaesthetic drugs are lacking.

However, there has been a shift in the demographics and number of patients who are undergoing PMC for RHD. Desnos et al. [3] published data from their centre in France showing that the average age of those undergoing PMC is rising, with the mean age going from 42.4 years (in 1987) to 55.4 years (in 2016). They also found that patients who were previously felt to have less favourable anatomy are now being offered PMC as experience with the procedure increases. It would therefore be useful to consider expanding this data set to older populations to see its effectiveness.

Finally, in 2012, Kang et al. [4] published their data showing that early PMC in asymptomatic moderate MS may outweigh the risks of early intervention. They carried out PMC in 244 consecutive asymptomatic patients (191 women, mean age 51 ± 11 years) who had moderate rheumatic MS. There were no procedure-related deaths and MVA was increased from 1.26 ± 0.11 to 2.07 ± 0.28 cm^2^ (*P* < 0.001). We feel it would be useful to expand Alcici et al.’s conscious sedation data to this moderate MS group, to see if it may further enhance the benefit to risk ratio of early intervention. This is because as surgery expands in developed countries these patients will also be operated on in larger numbers.

Thank you.
